# Sublingual misoprostol vs. oral misoprostol solution for induction of labor: A retrospective study

**DOI:** 10.3389/fsurg.2022.968372

**Published:** 2022-09-15

**Authors:** Mahdi Amini, Dag Wide-Swensson, Andreas Herbst

**Affiliations:** ^1^ Department of Obstetrics and Gynecology, Skåne University Hospital, Lund, Sweden; ^2^ Department of Obstetrics and Gynecology and Institution for Clinical Sciences, Lund University, Lund, Sweden

**Keywords:** human, pregnancy, induction of labour, misoprostol, sublingual, oral misoprostol solution, oral, retrospective

## Abstract

**Introduction:**

Induction of labor (IOL) is one of the most common obstetrical procedures, with an increasing rate. The prostaglandin E1 analogue misoprostol is frequently used as a primary method of labor induction. The optimal dose and route of administration is yet to be ascertained.

**Aim:**

To compare efficiacy and safety between a regimen of sublingually administered misoprostol and a regimen of orally administered misoprostol, with cesarean delivery as primary outcome.

**Methods:**

A retrospective study was conducted including women carrying a live, singleton fetus in a cephalic position with labor induced at >37 + 0 gestational weeks at Skåne University hospital, Lund, between January 1st 2013 to December 31st 2017. Data was obtained from computerized obstetrical charts.

**Results:**

Totally 2,404 women were included; 974 induced with sublingual misoprostol and 1,430 with oral solution. In primiparous women the cesarean delivery rate was lower in primiparous women induced with oral compared to sublingual misoprostol (20.5% vs. 28.6%, *p* < 0.001), whereas in parous women the rates did not differ significantly 4.9% vs. 7.5%; NS). The increased risk of caesarean remained after controlling for potential confounding factors (adjusted odds ratio 1.49 (1.14–1.95). Women induced with sublingual misoprostol had a shorter time to vaginal delivery when compared to oral solution (primiparous median 16.7 h vs. 21.7 h; *p* < 0.001, parous median 9.9 h vs. 13.3 h; *p* = 0.01), and a higher rate of vaginal delivery within 24 h (primiparas 77.7% vs. 63.3%, *p* < 0.001, parous 93.2% vs. 84.2%; *p* = 0.01).

**Conclusion:**

IOL with oral misoprostol solution was associated with a significantly higher vaginal delivery rate when compared to sublingual misoprostol, whereas sublingual misoprostol was associated with a significantly shorter time from induction to vaginal delivery. Oral administration is considered the most safe and efficient administration of misoprostol, although more studies are needed to find the optimal route and dosage of misoprostol for IOL.

## Introduction

Induction of labor (IOL) is one of the most common obstetrical interventions today. The rate of IOL in Sweden has risen from 13% in 2014 to 25% in 2020 (1). The most widely used cervical ripening methods are mechanical (balloon catheter and amniotomy) and pharmacological. The most used type of drugs in the case of an unfavorable cervix are prostaglandins which act both on the cervix and by potentiating uterine contractions (2).

Misoprostol is a prostaglandin E1 analogue, initially approved for the treatment and prevention of gastric ulcers from the use of non-steroidal anti-inflammatory drugs. It has been used for IOL for several years (3), and is now also approved in Sweden on this indication. Misoprostol has uterotonic effects and has also been used in gynecology for termination of pregnancy. The use of oral misoprostol for labor induction and treatment of postpartum hemorrhage is recommended by the World Health Organization (WHO) (3, 4).

Misoprostol is stable at room temperature and can be administered *via* several routes (oral, vaginal, sublingual and buccal) (5). A recent systematic review by the Cochrane institute indicated that IOL with oral misoprostol more often results in vaginal delivery than induction with vaginal dinoprostone, oxytocin or mechanical methods (6). The rate of vaginal birth was similar at induction with oral and vaginal misoprostol, although the rates of caesarean delivery for fetal distress as well as vaginal delivery within 24 h were higher with vaginal administration. There is more limited data regarding sublingual administration of misprostol to induce labor. However, in a network meta- and cost-effectiveness analysis of labor induction methods, including 19 randomized trials of sublingual or buccal misoprostol, Alfirevic et *al*. considered that “With a caveat of considerable uncertainty, titrated (low-dose) misoprostol solution and buccal/sublingual misoprostol had the highest likelihood of being cost-effective” (7).

During 2011–2014 misoprostol 50 µg sublingually every 4 h was the first-line method of labor induction at Skane University hospital, Lund. Since June 2014 the first line method has been oral misoprostol 20–40 µg administered every 2 h, derived from national guidelines recommending 25 µg every second hour (8).

The aim of this study is to compare the previous sublingual misoprostol regime to our current oral misoprostol solution regime with regards to efficacy (proportion of vaginal delivery, vaginal delivery within 24 h), maternal and neonatal morbidity.

## Methods

This is a retrospective cohort study based on patients referred for IOL between January 1st, 2013 and December 31st 2017 at the Department of Gynecology and Obstetrics, Skane University Hospital, Lund, Sweden.

Inclusion criteria were women carrying a live singleton fetus without major malformations, in cephalic position, without previous cesarean delivery, who had labor induced at a gestational age ≥37^+0^ weeks with misoprostol administered orally or sublingually. During the study period, a small number of patients were induced by other primary methods, such as vaginal prostaglandin E2 gel, slow-release vaginal insert of PGE2 or mechanical methods. Since the purpose of the study was to compare induction with misoprostol administered sublingually or orally, these patients were not included.

A structured protocol form for entering data was created. Data was obtained from electronic patient medical records and delivery charts (Obstetrix^™^) and subsequently transferred anonymized into a spreadsheet document (Microsoft Excel^™^). Demographic data included maternal age, body mass index (kg/m^2^), weight gain during pregnancy and gestational age. Gestational age was determined by ultrasound scanning at 11–13 or 18–19 weeks of gestation or, if not available, by last menstrual period. A modified Bishop's score (9), obtained through palpation immediately before induction, was retrieved from the medical records, as were indication for and method of induction. After inclusion, women were divided into two groups according to the method of induction. Inductions for premature rupture of membranes were performed when onset of regular contractions had not occurred at >24 h after membrane rupture. Inductions for postdate pregnancy included postterm pregnancies (≥294 days) and inductions for prolonged pregnancies at ≥287 days in women with risk factors. Hypertensive disorders of pregnancy was defined as those diagnosed with either preeclampsia or gestational hypertension. Fetal reasons for IOL included IUGR (intrauterine growth restriction), DFM (decreased fetal movements), oligohydramnion, and abnormal fetal heart rate patterns. Maternal reasons for IOL included diabetes (pregestational and gestational), suspected fetal macrosomia, chronic disease of the mother complicating pregnancy and other maternal causes.

The primary outcome was the proportion of patients who delivered vaginally, inversely expressed as the cesarean section rate. Secondary outcomes were induction to delivery interval, vaginal delivery within 24 h, postpartum hemorrhage (defined as blood loss >1,000 ml) Apgar score <7 at 5 min and umbilical artery pH < 7.10.

The two following regimes for IOL that were used during the study period were compared in this study:

Sublingual misoprostol (Cytotec, Pfizer Inc. New York, USA) was administered as giving a quarter of a 200 µg tablet sublingually at 4-hour intervals until favorable cervical ripening or labor was achieved, or for a maximum of 6 doses. The tablets were cut into quarters by scalpel. The patients were instructed to keep the tablet sublingually and to not swallow for at least 5 min.

The oral misoprostol solution was prepared by one 200 µg tablet of misoprostol being dissolved in 100 ml of water, yielding a concentration of 2 µg/ml. Women were then given the oral solution at 2-hour intervals. The first two doses were 10 ml (20 µg), and the following doses 20 ml (40 µg) of the oral solution every two hours until favorable cervical ripening or labor was achieved, or for a maximum of totally 12 doses.

### Statistical analysis

All data was entered into a SPSS file (SPSS 24 for Apple OS X, Chicago, IL, USA) for statistical analysis. Distributions of continuous variables were subject to the Kolmogorov-Smirnov test for normality. The Chi-square test was used for comparing categorical variables. The independent T-test was used to compare normally distributed continuous variables. The Mann-Whitney U test was used to compare non-normally distributed continuous variables. Analysis of variance (ANOVA) was used to compare mean values.

We wanted to analyze the association of cesarean delivery to the method of induction with adjustment for risk factors associated with cesarean (8). The risk factors adjusted for were maternal age (<40 years or >40 years), parity (primi- or multiparous), gestational age <41 weeks or ≥41 weeks), indication for induction [premature rupture of membranes (PROM), postdate, hypertensive disorders of pregnancy, maternal, fetal and non-medical reason for IOL] and Bishop's score. We considered that adjustment for year of delivery was not possible since the method of induction was too closely related to year of delivery. The crude (unadjusted) association of each risk factor and cesarean section was first calculated. Binary logistic regression was then used to adjust for the association between the risk factors and cesarean delivery to the method of induction. The Hosmer and Lemeshow test and Hoffmeyer Goodness of fit test was used. The associations are presented as odds ratios (OR) with 95% confidence intervals (CI). A 95% confidence interval not including 1.0, and a *p*-value of <0.05 was considered statistically significant.

The study was approved by the regional ethics committee in Lund, file record: 2018/546.

## Results

### Baseline characteristics

**Figure 1:**
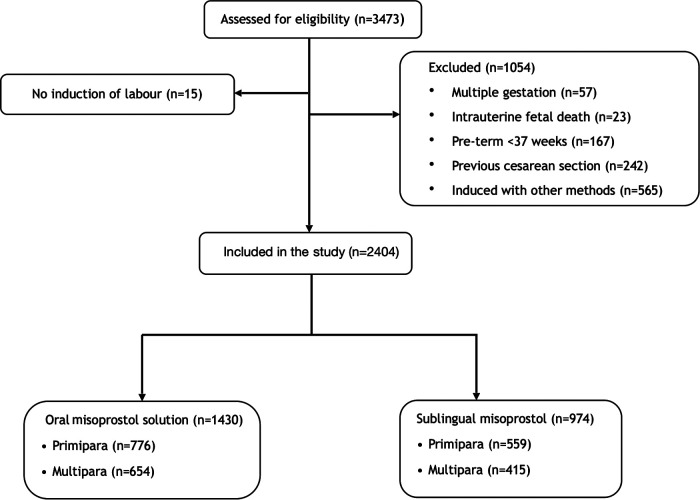
Flow chart of the included patients.

During the observed period, a total of 3,473 women were induced of whom 2,404 fulfilled the inclusion criteria and were included in the study (Figure 1). The total induction rate during the study period was 18.6%. Baseline characteristics are shown in Table 1. There were no significant differences between women induced with the oral misoprostol solution and sublingual misoprostol with regards to age, BMI, gestational age, weight gain or Bishop´s score (Table 1). In primiparas, there was a significant difference in the indication for induction between the two groups (*p* = 0.006). The most common indication for induction in primiparas were prolonged pregnancy (34%), PROM (21%) and fetal reason (17.5%). More primiparas were induced because of PROM and fetal indications in the oral solution group. In multiparas, there was also a significant difference in the indication for induction (*p* = 0.026). The most common reasons for inducing labor in multiparas were fetal reason (28%), postdate (20%) and non-medical (17.5%). More multiparas were induced on fetal indications in the oral solution group.

**Table 1:** Demographic characteristics.

	Primipara			Multipara		
	Oral solution *n* = 776	Sublingual *n* = 559	*p*-value	Oral solution *n* = 654	Sublingual *n* = 415	*p*-value
Age^a^	29.8 (5.0)	29.9 (4.9)	*p = 0.697*	32.8 (4.7)	32.6 (5.1)	*p* = 0.501
BMI^a^	31.1 (5.0)	30.8 (5.1)	*p = 0.429*	30.8 (4.9)	31.0 (5.5)	*p* = 0.681
Gestational age (days)^a^	282 (10)	283 (10)	*p = 0.098*	278 (11)	278 (10)	*p* = 0.434
Weight gain (kg)^a^	15.6 (6.2)	15.3 (6.2)	*p = 0.492*	13.7 (6.3)	13.5 (6.0)	*p* = 0.752
Bishop score^a^	3 (2)	3 (2)		3 (2)	3 (2)	
Indication for induction^b^			*p *=* 0.006*			*p* = 0.026
Postdate	248 (32.0)	206 (36.9)		133 (20.3)	78 (18.8)	
PROM	182 (23.5)	102 (18.2)		97 (14.8)	51 (12.3)	
Fetal reason	151 (19.5)	83 (14.8)		197 (30.1)	107 (25.8)	
Hypertensive disorder	115 (14.8)	89 (15.9)		46 (7.0)	28 (6.7)	
Maternal reason	43 (5.5)	50 (8.9)		71 (10.9)	74 (17.8)	
Non-medical reason	37 (4.8)	29 (5.2)		110 (16.8)	77 (18.6)	

^a^ Values are mean (± SD).

^b^ Values are frequencies (%).

PROM, premature rupture of membranes; BMI, Body mass index.

### Primary outcomes

Primary and secondary outcome parameters are shown in Table 2. In primiparas, the rate of cesarean delivery was significantly lower after induction with oral (20.5%) than with sublingual misoprostol (28.6%, *p* < 0.001). Among multiparas the cesarean delivery rate in the oral solution group (2.1%) was insignificantly lower than in the sublingual group (7.5%, *p* = 0.065). The higher risk of caesarean among women induced with sublingually administered misoprostol remained after controlling for potential confounding factors (Table 3).

**Table 2:** Primary and secondary outcomes.

	Primipara			Multipara		
	Oral solution *n* = 776	Sublingual *n* = 559	*p*-value	Oral solution *n* = 654	Sublingual *n* = 415	*p*-value
Mode of delivery			*p = 0.001*			*p = 0.055*
Vaginal	510 (65.7)	316 (56.5)		608 (93)	377 (90.8)	
Instrumental	107 (13.8)	83 (14.8)		14 (2.1)	7 (1.7)	
Cesarean	159 (20.5)	160 (28.6)		32 (4.9)	31 (7.5)	
Time from induction to vaginal delivery (h)	21.7 (14.6–30.8)	16.7 (10.7–25.1)	*p < 0.001*	13.3 (9.6–20.2)	9.9 (7.1–14.8)	*p = 0.001*
Vaginal delivery <24 h	390 (63.2)	310 (77.7)	*p < 0.001*	524 (84.2)	358 (93.2)	*p = 0.001*
PPH >1,000 ml	91 (11.7)	48 (8.6)	*p = 0.064*	44 (6.7)	28 (6.7)	*p = 0.982*
Apgar score <7 at 5′	11 (1.4)	13 (2.3)	*p = 0.218*	7 (1.1)	8 (1.9)	*p = 0.243*
Cord artery pH < 7.10^a^	39/542 (7.2)	14/241 (5.8)	*p = 0.473*	18/439 (/4.1)	14/154 (9.1)	*p = 0.02*

Values are numbers (%) except for time from induction presented as median (interquartile range).

PPH, postpartum haemorrhage.

^a^ Percentages of pH < 7.10 among neonates with obtained umbilical cord blood samples.

**Table 3:** Odds ratio for cesarean delivery adjusted for various risk factors.

Risk factor	CS/Total (%)	OR unadjusted (95% CI)	OR adjusted (95% CI)
Maternal age			
<30 years	145/943 (15)	Ref.	Ref.
>30 years	237/1,461 (16)	1.07 (0.85–1.33)	1.46 (1.11–1.93)
<40 years	350/2,262 (15)	Ref.	Ref.
>40 years	32/142 (22)	1.59 (1.05–2.39)	2.53 (1.46–4.36)
Parity			
Multiparous	63/1,069 (6)	Ref.	Ref.
Primiparous	319/1,335 (29)	5.01 (3.77–6.66)	6.04 (4.24–8.60)
Gestational age (weeks)			
<41 + 0	213/1,597 (13)	Ref.	Ref.
>41 + 0	169/807 (21)	1.78 (1.38–2.15)	1.36 (0.89–2.08)
Indication for induction			
PROM	25/253 (10)	Ref.	Ref.
Non-medical	47/432 (11)	0.90 (0.54–1.50)	1.36 (0.71–2.63)
Maternal reason	31/238 (13)	1.22 (0.75–2.00)	1.83 (1.03–3.25)
Hypertensive disorders of pregnancy	81/538 (15)	2.06 (1.35–3.15)	1.82 (1.08–3.05)
Fetal reason	56/278 (20)	1.45 (0.99–2.13)	1.94 (1.22–3.09)
Postdate	142/665 (21)	2.22 (1.56–3.17)	2.03 (1.19–3.46)
Bishop score			
>5	16/182 (9)	Ref.	Ref.
<5	366/2,222 (16)	2.05 (1.21–3.46)	2.55 (1.30–5.00)
Body mass index			
<35	216/1,487 (14)	Ref.	Ref.
>35	77/373 (20)	1.53 (1.15–2.04)	1.52 (1.12–2.09)
Method of induction			
Oral solution	191/1,430 (13)	Ref.	Ref.
Sublingual	191/974 (20)	1.58 (1.27–1.97)	1.49 (1.14–1.95)

PROM, premature rupture of membranes.

### Secondary outcomes

Primiparas induced with sublingual misoprostol had a shorter time to vaginal delivery when compared to oral solution (*p* < 0.001; median 16.7 h vs. 21.7 h). The proportion of primiparous women delivered vaginally within 24 h was higher in the sublingual group (77.7% vs. 63.3%, *p* < 0.001). Also in parous women, the time from induction to vaginal delivery was shorter in the sublingual group (median 9.9h) than in the oral group (median 13.3 h, *p* < 0.001), and the proportion of women delivered vaginally within 24 h higher in the sublingual group (93.2% vs. 84.2%, *p* < 0.001). There were no significant differences between the two groups with regards to PPH and 5-minute Apgar score <7. In parous women, the rate of cord artery pH < 7.10 was significantly higher in the sublingual group (9.1% vs. 4.1%, *p* = 0.02).

## Discussion

The main findings in this study were that, in primiparous women, IOL with oral misoprostol 20–40 µg every second hour resulted in a higher rate of vaginal delivery than induction with sublingual misoprostol 50 µg every 4 h, whereas the latter regime resulted in shorter labor to delivery intervals in both primiparous and parous women. The higher risk of caesarean delivery at induction with sublingually administered misoprostol remained after controlling for potential confounding factors.

Our rates of cesarean delivery were similar to the rates in other studies in which oral misoprostol solution was used (10–12), which might support external validity for the higher rate of vaginal delivery with oral administration of misoprostol. The finding is also in accordance with the Cochrane review reporting that induction with low dose oral misoprostol is associated with a higher chance of vaginal delivery than induction with vaginal dinoprostone, oxytocin or mechanical methods (6).

By contrast, the proportion of women delivered vaginally within 24 h with sublingual misoprostol was significantly higher than with the oral misoprostol solution, a result which was consistent for both primiparous and multiparous women, and the induction to delivery intervals were shorter in inductions with sublingual misoprostol. Time from induction to delivery is a factor that might affect maternal birth experience, but studies do not support that time to delivery interval is an important factor for maternal satisfaction (13–15).

Sublingual misoprostol for IOL has been compared to vaginal misoprostol in a review by Souza et al., including 5 studies with totally 740 women (16). There were no significant differences regarding delivery or neonatal outcomes. In a Cochrane review from 2004, studies comparing oral with sublingual misoprostol did not show significant differences in rates of caesarean or vaginal delivery within 24 h, but the included number of patients were small (17). Although there was insufficient data regarding the safety for sublingual misoprostol, the authors concluded that sublingual misoprostol was as least as effective as vaginal (16), and oral (7, 17) misoprostol. In line with our results, a study that compared sublingual misoprostol to oral misoprostol given every four hours showed a significantly higher proportion of women delivered vaginally within 12 h when given sublingual misoprostol (18).

Most of the literature regarding sublingual misoprostol for IOL are comparisons to vaginal misoprostol, most probably because of the similar pharmacokinetic profile. It bypasses the first-pass metabolism by the liver which leads to a shorter time to maximum drug concentration (Tmax), a. higher maximum serum concentration (Cmax) and a larger area under the curve (AUC) when compared to oral administration (5, 19). Higher plasma concentrations of misoprostol after sublingual than after oral administration might explain both shorter induction to delivery intervals and a higher risk of caesarean delivery, since higher concentrations are likely to lead to higher uterine activity, with an increased risk of hyperstimulation. Our finding of a higher rate of neonates with low cord artery pH among multiparous women induced by sublingual misoprostol is consistent with this assumption. We therefore consider that 50 µg misoprostol administered sublingually every 4 h may be a too high dose for maternal and fetal safety. Today we use the same dose regimes generally on different indications in women with different risk profile, parity and body weight. Among emergency cesarean delivery for fetal distress in the sublingual group, we saw a relatively larger proportion of patients who were induced because of IUGR or oligohydramnion. Some groups undergoing IOL are likely to be more sensitive to a high uterine activity, and a more careful approach is warranted to safely deliver these patients vaginally.

Misoprostol has a relatively short half -life, and therefore a two-hourly regimen might fit it's pharmacokinetic properties optimally (20). Because of the variation of effects on women receiving the same dose, a titrated oral misoprostol regime tailored to the specific patient and indication for IOL may be a focus of future studies.

### Strengths and limitations

A strength of our study is a relatively large number of included women induced with misoprostol either administered as an oral solution or sublingually. There is, to our knowledge, no prior single study published including such a large number of women induced with a sublingual regime. A limitation of our study is the retrospective design, and that the two regimes were used during two different time periods. However, we did adjust for potential causes of bias, including parity and indication for induction. In the adjusted model we did not include year of delivery because the method of induction was so too closely related to year of delivery to make adjustment possible. Although the cesarean delivery rate in labors induced with misoprostol declined from 20,9% during 2013 when sublingual misoprostol was the primary method, to 11,8% during 2016–17 when oral misoprostol was the primary method, a similar trend was not seen for all laboring women. By contrast, the total emergency cesarean delivery rate was 10,7% in 2013 and 10,9% in 2016–17. Thus, we consider it unlikely that the lower cesarean section rate for inductions with oral misoprostol would have to do with other general changes in obstetric practice during these years.

## Conclusion

The cesarean delivery rate was significantly higher in primiparas induced with the sublingual regime, and the rate of spontaneous vaginal delivery significantly higher in those induced with low dose oral misoprostol. A significantly lower risk of cesarean delivery remained after adjusting for confounding factors. Misoprostol given the sublingual route had a significantly shorter time from induction to delivery when compared to the oral misoprostol solution.

## Data Availability

The raw data supporting the conclusions of this article will be made available by the authors, without undue reservation.
